# Artificial Intelligence in Predicting Systemic Complications From Retinal Findings: A New Frontier in Precision Medicine

**DOI:** 10.7759/cureus.114560

**Published:** 2026-08-15

**Authors:** Regalla Kinnera, Tameema Kauser, Manfredi Giammanco, Hassen A AlHussen, Arvin Khachikian, Seeme Rukh, Mohamed Alansaari, Wasib Ali

**Affiliations:** 1 Anesthesiology, Osmania Medical College, Hyderabad, IND; 2 Ophthalmology, Osmania Medical College, Hyderabad, IND; 3 Medicine, University of Palermo, Palermo, ITA; 4 Critical Care Medicine, Dr. Sulaiman Al Habib Medical Academy, Riyadh, SAU; 5 Internal Medicine, St. George’s University, True Blue, GRD; 6 Medicine, Malik Haider Hospital, Gujrat, PAK; 7 General Internal Medicine, Royal College of Surgeons in Ireland, Dublin, IRL; 8 Internal Medicine, Dow International Medical College, Karachi, PAK

**Keywords:** artificial intelligence, cardiovascular disease, deep learning, diabetic retinopathy, fundus photography, neurodegenerative diseases, optical coherence tomography, retinal biomarkers, retinal imaging, systemic disease prediction

## Abstract

Innovations in retinal imaging technologies and growing evidence from retinal imaging of systemic and neurodegenerative diseases have begun to explore the utility of retinal imaging in diagnosing these conditions. Since the retina shares embryological origins with the central nervous system and reflects systemic microvascular characteristics, it is well positioned for noninvasive observation of patients' systemic and neural health. Moreover, accessibility of retinal imaging has improved with the increasing number of ophthalmology clinics. Rapid improvements in various deep learning (DL) tools have also catalyzed the automation of retinal imaging analysis. Systems that utilize DL for retinal imaging are being developed to assist with disease recognition, clinical judgment, and prognostic assessment of systemic health. Various imaging modalities are being integrated with existing genomic and clinical data to estimate an individual’s predisposition to certain conditions. Contrary to many existing reviews, the objective of this review is to synthesize the most recent clinical and technological evidence on DL-based diagnostic systems for retinal imaging, with a focus on how different network architectures and their combinations have been developed, validated, and applied across systemic disease detection and prediction. Specifically, this review examines the datasets, model validation approaches, and automated diagnostic systems reported in recent literature. It discusses the extent to which these advancements address existing barriers toward real-time diagnostic application across clinical disciplines. Integrating retinal imaging with DL is an innovative and promising approach to precision medicine and health risk reduction.

## Introduction and background

Retinal structural and vascular parameters are commonly studied in cerebrovascular and central nervous system (CNS) diseases [1]. There are many developmental processes shared among the retina, the CNS, the cardiovascular system, and their microcirculation [2,3]. Some studies conducted in subjects with internal carotid stenosis have shown that retinal structure and vascular dynamics, such as choroidal thickness, play a predictive role in assessing carotid stenosis severity [4-6]. Standard diagnostic exams for retinal diseases have limitations, including pupil dilation, side effects of fluorescein injection (nausea, feeling faint, and more serious complications such as breathing problems or even life-threatening complications), and a narrow acquisition field. While newer imaging modalities have helped reduce some of these acquisition barriers, the resulting increase in image volume has created a need for consistent, efficient, and reproducible interpretation [7-9]. This is where AI plays a central role; rather than addressing acquisition limitations directly, AI enhances the management and identification of systemic disease complications by enabling automated feature extraction and risk prediction from retinal images at a scale and consistency not achievable through manual grading alone [10].

A substantial number of studies have examined the role of AI in diagnosing systemic diseases using retinal imaging [11]. To investigate the diagnostic accuracy of AI for grading atherosclerotic retinopathy, Gao et al. developed a four-channel convolutional neural network (CNN) trained on fundus retinal images [12]. In 2021, Rim et al. introduced RetiCAC, an AI tool trained on 216,152 retinal photographs to estimate the coronary calcium score, which is useful for risk stratification of cardiovascular disease (CVD) events [13].

This study explores how AI-driven analysis of retinal images could aid in the earlier detection of cardiovascular, metabolic, and systemic diseases. Predicting systemic diseases with AI guidance plays a key role in helping clinicians make prevention more personal, precise, and accessible. AI has also assumed an increasingly prominent role in research and scholarly publishing. This growing use warrants clear and transparent reporting of AI involvement.

## Review

Methods

This study employed a narrative literature review using secondary data from published peer-reviewed articles. Relevant studies were identified in databases such as PubMed and Google Scholar using keywords including AI, retinal imaging, deep learning (DL), systemic disease prediction, and precision medicine. The collected literature was critically analyzed to summarize current evidence, technological advances, clinical applications, and research gaps.

Retina as biomarker for systemic health

Microvascular and Neural Parallels Between the Retina and Systemic Organs

The retina enables noninvasive in vivo examination of vascular and neural structures, giving insight into systemic health. As extensions of the CNS and reflections of systemic vascular function, retinal features can serve as biomarkers for a wide range of physiological conditions [14]. Subtle changes in the retinal microvasculature often mirror similar changes in other organs. An emerging hypothesis is that microvascular disease is frequently a systemic process. For example, microvascular dysfunction in the heart commonly coexists with similar microvascular issues in the brain, kidneys, retina, and lungs [15]. Thus, indicators of microvascular changes in the retina may suggest similar damage in the coronary arteries, cerebral circulation, or renal microvessels.

As the retina is an extension of the CNS, it also offers insights into neural health. Changes in the retinal nerve fibers and cells may reflect parallel changes in the brain's neurons. For example, a thinning of the retinal nerve fiber layer, detectable via optical coherence tomography (OCT) scans, has been linked to both cognitive decline and neurodegenerative conditions such as Alzheimer's. Similarly, the retina’s microvasculature shares similarities with brain microvessels, so retinal microvascular abnormalities may indicate cerebrovascular issues. Studies have shown that changes in retinal vessel caliber or shape correlate with cardiovascular risk factors and can even predict future events such as stroke and myocardial infarction [16].

Retinal Signs Linked to Systemic Conditions: Arteriolar Narrowing, Arteriovenous Nicking, Hemorrhages, and Microaneurysms

Retinal exam findings such as arteriolar narrowing, arteriovenous (AV) nicking, hemorrhages, and microaneurysms are associated with systemic conditions, including hypertension and diabetes, as well as with diabetic retinopathy (DR). These reflect microvascular damage from high blood pressure or high blood sugar. For example, chronic hypertension causes the small retinal arteries to constrict and thicken, resulting in generalized arteriolar narrowing on an eye exam. It also leads to AV nicking, where an artery crosses over a vein and compresses it, a sign of arterial stiffening. Retinal hemorrhages and microaneurysms indicate advanced microvascular damage from uncontrolled hypertension or diabetes. Retinopathy signs are classified as mild or moderate. Mild signs (slight arteriolar narrowing, AV nicking) correlate weakly with cardiovascular events despite links to chronic hypertension. Moderate signs (retinal hemorrhages, microaneurysms) strongly associate with severe outcomes like stroke and heart failure [17]. Retinal hemorrhages exhibit a quantitative correlation with white matter hyperintensities, which indicate cerebral small vessel disease, with an odds ratio (OR) of 2.23. Additionally, AV nicking is associated with lacunar infarcts, with an OR of 1.70 [18].

Hypertension and Cardiovascular Disease

Hypertension and CVD are major causes of morbidity and mortality worldwide. In this context, retinal imaging offers a noninvasive method to assess microvascular changes associated with these conditions. Research has shown that retinal biomarkers such as arteriolar narrowing and arterial stiffness are strongly associated with hypertension and CVD. Both retinal vessel perfusion and blood flow are influenced by arterial stiffness and provide insight into structural and functional alterations of systemic vasculature [19].

Stroke

While stroke is a major cause of disability and death globally, the use of retinal imaging offers a glimpse into the microvascular changes of the brain, allowing for the assessment of stroke risk. Several retinal biomarkers are associated with stroke, including wider retinal venules, increased arteriolar tortuosity, and retinal emboli [20].

Diabetes and Diabetic Retinopathy

More than 422 million people worldwide are affected by diabetes mellitus (DM), a major cause of morbidity and mortality. With DR becoming one of the most frequent complications of DM, newer developments in AI have expanded the application of retinal imaging for the identification of other DM-related complications, such as neuropathy and nephropathy. Retinal biomarkers such as nerve fiber layer thinning, microvascular rarefaction, and capillary nonperfusion have been strongly correlated with neuropathy and nephropathy in diabetes, highlighting compromised systemic microvascular integrity [21].

Alzheimer’s Disease and Cognitive Decline

Neurodegenerative diseases such as Alzheimer’s disease (AD) and dementia have become a growing public concern with few early diagnostic tools at our disposal. Retinal imaging provides a noninvasive and inexpensive tool to study early neurodegenerative changes. Cognitive decline can be measured using retinal biomarkers such as thinning of the nerve fiber layer, reductions in vessel density, and fractal dimension, which can map the early stages of AD progression [22].

Chronic Kidney Disease

Chronic kidney disease (CKD) affects millions of people worldwide and is associated with an increased risk of cognitive impairment and CVD. Retinal imaging detecting microvascular damage parallels the changes occurring in the kidneys. According to findings from the Chronic Renal Insufficiency Cohort study, almost 49% of participants with CKD had retinal signs of microvascular disease, including retinopathy and changes in vessel caliber. These retinal findings provided independent information about kidney function, even after accounting for traditional CKD risk factors such as eGFR [23].

Traditional Retinal Evaluation Methods: Strengths and Limitations

Retinal imaging provides several benefits for evaluating systemic health. Imaging modalities such as OCT and fundus photography are widely available in ophthalmology clinics, enhancing accessibility and reducing the need for patients to be referred to specialized clinics. Additionally, retinal imaging, compared to invasive methods such as fluorescein angiography (FA) and biopsies, is relatively inexpensive, painless, and noninvasive, and it offers high sensitivity by identifying subtle microvascular changes even at an early stage [24].

Conventional imaging techniques also have significant limitations. One such limitation is that conventional imaging modalities may lack the specificity to differentiate among various systemic conditions. Systemic diseases such as diabetes, hypertension, and various CVDs present with similar retinal microvascular changes, making it difficult to distinguish between them when using standalone conventional analysis [24]. Moreover, manual interpretation is often required, which creates the potential for subjective error and variability [24]. While these imaging modalities are promising tools for assessing systemic health in a cost-effective, noninvasive manner, they are not yet fully integrated into broader systemic health assessments, thereby limiting their use in primary care settings [19].

Evolution of AI in retinal imaging

Progression to Deep Learning

DL applications in ophthalmology have been extensively studied since 2016. Early well-known comparative studies on DR were conducted by Abràmoff et al. and Ting et al. They found that CNN-based lesion detectors within a hybrid architecture achieved significantly better performance in detecting referable DR, with a sensitivity of 96.8% and a specificity of 59.4% [25,26]. Furthermore, Gulshan et al. reported similar findings using a larger dataset [27]. Two large macula-centered retinal fundus image datasets were used: EyePACS (9963 images from the USA) and Messidor-2 (1748 images from France). Fifty-four US board-certified ophthalmologists graded those images using the International Clinical Diabetic Retinopathy Scale, achieving sensitivities of 97.5% and 96.1%. The study reported that sensitivity and specificity can be tuned to meet requirements in specific clinical settings, such as high sensitivity in screening.

Kermany et al. evaluated a limited model using 1,000 randomly selected uncategorized images in a diagnostic AI system. After reviewing images with choroidal neovascularization and images with diabetic macular edema (DME), AI was able to distinguish cases requiring urgent referral for definitive anti-VEGF treatment, with an accuracy of 93.4%, a sensitivity of 96.6%, and a specificity of 94.0% [28]. Despite the limited availability of large amounts of data, the homogeneity of data within a given population, and poor image quality, the incorporation of AI in ophthalmology is progressing [29].

Foundation Models

The term "foundation models" was coined by the Stanford Institute for Human-Centered Artificial Intelligence [30]. These are transfer learning and DL models trained on broad data at scale and adaptable to a wide range of downstream tasks. These models have certain advantages, including improved label efficiency, enhanced generalizability, and reduced computational requirements during fine-tuning. In particular, there is improved performance among minority ethnic groups, which is a key concern when implementing models trained using traditional approaches [31].

With the advent of new AI techniques, recent advances in retinal imaging have contributed to preventive medicine. A cohort study of 417 patients by Dow et al., using multiple datasets, analyzed volumetric spectral-domain OCT images with a CNN as an image-analysis AI technique. At a high-specificity operating point, simulated clinical trial recruitment was enriched for patients progressing to GA within one year by 8.3- to 20.7-fold [32].

Convolutional Neural Networks

CNNs are used to implement most computer vision algorithms and are extensively employed in retinal disease research. A CNN consists of multiple layers of feature transformations, in which the underlying feature representation of the original data is gradually transformed into a higher-level representation [33]. Other architectures used include the fully convolutional network, the U-shaped network (U-Net), and other hybrid computational methods. Lin et al. compared these models in the retinal fluid segmentation literature and found that the SEADNet model achieved the best overall segmentation results [34].

Transformers

In 2017, Transformers, "a type of neural network architecture," initially developed for natural language processing, showed promise in handling high-dimensional data. Transformers addressed limitations associated with sequential processing through two key innovations: positional encoding and attention mechanisms. These innovations led to substantial improvements in parallelization and the ability to scale training to large datasets. The Transformer architecture was introduced at the 31st International Conference on Neural Information Processing Systems [31].

Generative Adversarial Networks

Another model crucial to the future of AI in detecting ophthalmologic diseases is a generative adversarial network (GAN). GAN techniques are typically used to segment retinal vessels from fundus photographs. Other applications of GANs include segmentation, data augmentation, denoising, domain transfer, super-resolution, post-intervention prediction, and feature extraction. Compared with the CNN model, the GAN model is more complex. The generator uses the source image to produce a synthetic approximation in the new modality, and the discriminator then attempts to distinguish the synthetic image from the corresponding real image in the new modality [35,36].

One particular GAN method, pix2pixHD, used by Liu et al., generates and evaluates individualized post-therapeutic OCT images that predict the short-term response to anti-vascular endothelial growth factor therapy in typical neovascular age-related macular degeneration (AMD) from pretherapeutic images. This study concluded that 92% of the synthetic OCT images had sufficient quality for further clinical interpretation [37]. Given these findings, GANs have tremendous potential in ophthalmology. Another application is to remove blood vessel shadows from OCT images of the optic nerve head using DeshadowGAN, thereby decreasing the intralayer contrast across all tissue layers [38].

AI in imaging modalities

AI in Fundus Photography

Fundus photography is a cornerstone of ocular diagnostics, offering a noninvasive window into the health of the retina and systemic vasculature. With the rise of AI, particularly DL, fundus imaging has transformed into a powerful modality for automated disease detection, clinical decision support, and systemic health prediction [39,40].

The use of AI for fundus imaging began with the digitization of retinal photographs in the late 20th century. Methodologies developed in the 20th century primarily directed early AI toward blood vessel segmentation and microaneurysm identification, using human-designed mathematical shapes and features [40]. Baudoin et al. were among the earliest groups to identify microaneurysms in digital angiographic images and to commence AI-augmented screening of DR [41].

The mid-2010s marked a major milestone with the incorporation of machine learning (ML) and DL models. Using large-scale image datasets, these models were the first to detect a multitude of pathological features, including hemorrhages, exudates, and neovascularization [42]. An initiative established by DeepMind and Moorfields Eye Hospital was the first to use CNNs on fundus and OCT images. This project was the first to identify and classify DR and wet AMD at an expert level [43].

Supervised learning models gained popularity for classifying disease severity using labeled fundus photographs. These systems provided classification and lesion localization, thereby enhancing interpretability and supporting clinical validation [43]. Several AI-based systems have since received regulatory clearance for clinical use. Notable examples include IDx-DR (Digital Diagnostics), EyeArt (Eyenuk), and VoxelCloud Retina [44]. Modern AI models integrate data from multiple modalities (fundus images, OCT, and FA) to assess not only ocular disease but also systemic risk factors for cardiovascular health. For instance, AI models such as Deepeye use OCT to suggest AMD treatments based on fluid dynamics [45]. Attention-gated CNNs have been used to detect leakage in central serous retinopathy with greater accuracy than human graders. Style transfer and image registration networks have enhanced AI's strength in heterogeneous imaging conditions [42].

The study by Zekavat et al. used DL on over 97,000 UK Biobank fundus images to segment retinal vessels and quantify vascular fractal dimension and density, linking these features to over 1,800 diseases through phenome- and genome-wide analyses. Their findings show that reduced vascular complexity is associated with a higher risk of systemic and ocular diseases and identify 20 novel genetic loci, establishing AI-enhanced fundus imaging as a powerful tool for multi-disease risk prediction [46].

AI in Optical Coherence Tomography

The application of AI with OCT has matured from elementary image processing to advanced, multifaceted clinical decision support systems capable of performing multiple tasks. This shift has resulted from a combination of developments in DL and the growing recognition of OCT as an important tool for assessing both ocular and systemic health [47].

The earliest applications of AI in OCT focused on segmentation and feature computation. One prominent example is the work of Li et al., who developed a segmentation model to identify 10 retinal layers in both healthy eyes and eyes affected by AMD or DR [48]. These early models automated a previously labor-intensive manual process. With time, other models followed. Some of these models used DL to quantify choroidal thickness in high myopia, while others used DL to segment ONH structures. According to Panda et al., strong Dice coefficient performance proved the efficacy of these models and suggested the applicability of AI to structural OCT analysis [49]. Researchers have attempted to solve the problem of limited manual annotation as the demand for scalable AI solutions has grown. Devalla et al. introduced ONH-Net, a three-dimensional, segmentation, annotation-free framework that shows consistent results across multiple OCT devices [50]. This development was important for generalizability and for potential use in clinical settings with multiple imaging techniques. The application of advanced techniques, such as few-shot learning and GANs, has expanded the scope of AI in diagnosing atypical ophthalmic disorders. These methods increased the potential of learning from small datasets, which is especially important for rare diseases [51].

At the same time, the creation of multitask and weakly supervised models enabled the simultaneous classification of multiple conditions, bringing AI applications closer to real-world screening and clinical settings. Notably, models developed on longitudinal OCT datasets have shown the potential to predict disease course, identify treatment responders, and anticipate which treatments may be required, significantly changing the role of AI in diagnosis and longitudinal care planning [47,51].

The models developed by Kermany et al. and de Fauw et al. have played an important role in assisting with clinical triage and decreasing clinician workload. These models provide examples of AI's ability to assist with and/or reduce the complexity of clinical decision-making. However, the actual use of these models will require significant integration with existing clinical workflows and rigorous assessment across relevant patient populations [39].

AI-enhanced OCT analysis has, in recent iterations, extended beyond merely ophthalmological conditions. The small vessels of the retina have increasingly been valued as indicative of systemic disease. For instance, Lopez-Dorado et al. used AI to identify early-stage multiple sclerosis, achieving perfect sensitivity and specificity. In a similar vein, Wisely et al. combined multiple forms of retinal imaging and AI to forecast the presence of AD. Together with findings from other studies, this demonstrates OCT's ability to serve as a noninvasive indicator of a wide range of neurological and vascular diseases [16].

AI in Fundus Fluorescein Angiography

The application of AI in fundus FA (FFA) is evident in several areas, including lesion detection, diagnostic assistance, and automated clinical reporting. Many of these applications rely on DL and have improved diagnostic accuracy, workflow efficiency, and consistency in assessing retinal vascular disorders [52].

**Figure 1:**
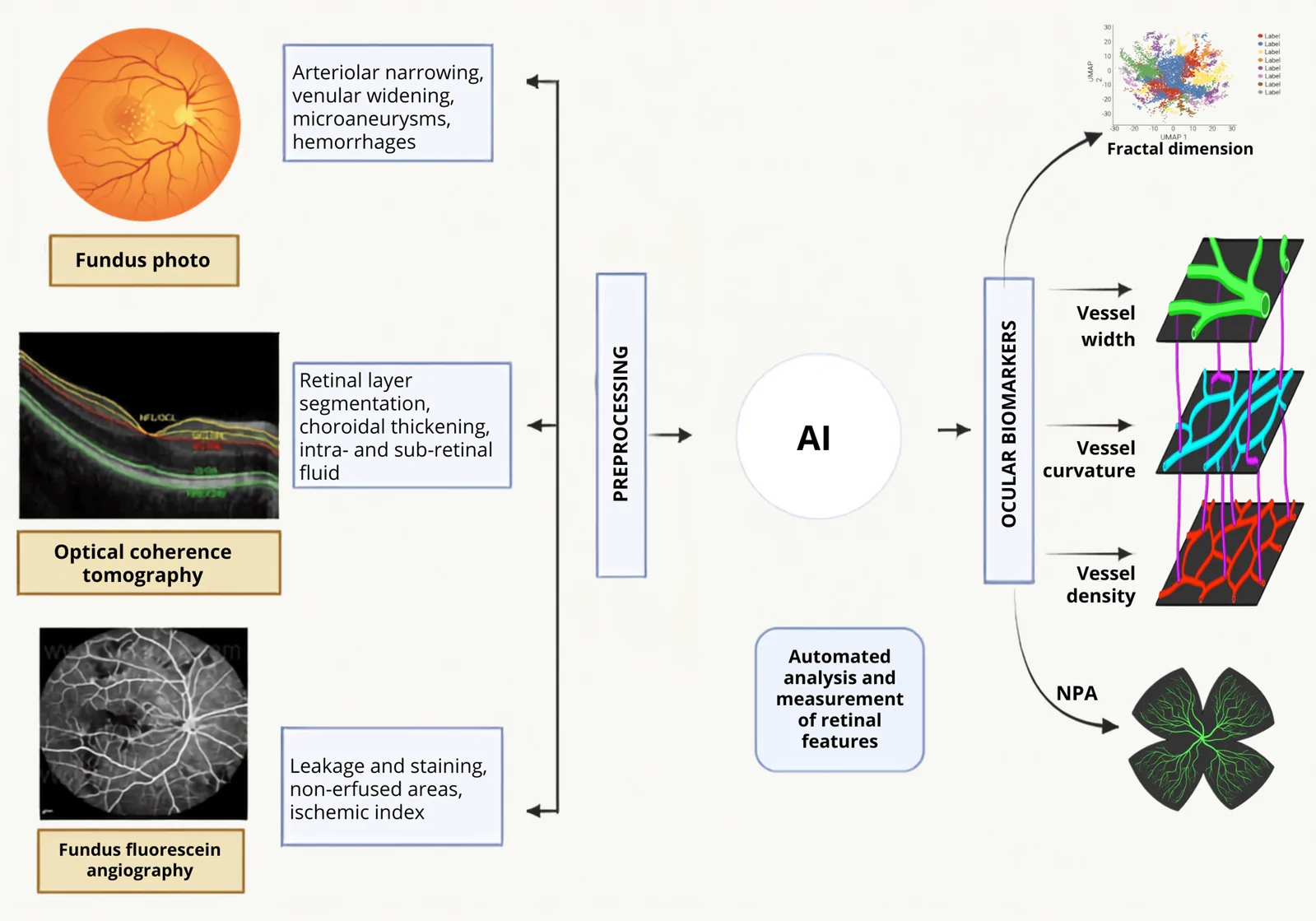
DL models in retinal image analysis DL: deep learning, AI: artificial intelligence, NPA: non-perfusion area Image Credit: Giammanco M (2025) using Science Suite Inc. (BioRender; Toronto, Ontario, Canada)

AI tools intended for automated lesion detection have primarily focused on detecting and segmenting various pathological cues in FFA images. These include microaneurysms, non-perfusion areas, vascular anomalies, and fluorescein leakage. One example is the work of Sun et al., who developed a multi-path cascade U-Net to preserve the integrity of fine vascular structures across different perfusion stages. The results from the internal and external datasets demonstrate the robustness of the design and its applicability to quantitative measurement of vascular structures and to the temporal assessment of related pathologies [53]. Li et al. made a significant breakthrough by incorporating a CNN-attention framework into multimodal datasets comprising color fundus photographs (CFPs) and FFAs, demonstrating impressive performance for cross-modality diagnosis and bolstering AI's capability for reproducible and scalable diagnostic decision support (Figure 1) [52].

The most recent frontier in this evolution is the development of AI models capable of generating comprehensive diagnostic reports from FFA sequences. These systems aim to reduce reliance on specialist interpretation while enhancing clinical output. For instance, Chen et al. proposed a multimodal framework that integrates the bootstrapping language-image pretraining framework with the LLaMA 2 language model to produce free-text diagnostic reports and support interactive Q&A functionalities [54]. Complementing this, Zhao et al. developed "Ai-Doctor," a multitask system trained on over 24,000 FFA images to perform disease classification, ischemic index computation, and segmentation of pathological regions. The system’s output was tailored to assist in therapeutic decision-making, including laser intervention planning [55].

Emergence of retinal fingerprinting and personalized risk profiling

Retinal vascular patterns are unique to each individual and remain largely unchanged throughout life, making them ideal for secure biometric authentication, as demonstrated by Köse and İkibaş in their development of a retinal identification system achieving over 95% accuracy while tolerating variations in scale, rotation, and translation. Simultaneously, advances in retinal imaging, particularly OCT and OCT angiography (OCTA), enable high-resolution visualization of retinal microvascular structures [56].

These technologies have been shown to correlate with systemic CVD risk factors, such as hypertension and CKD, enabling clinicians to assess risk noninvasively and at an individualized level. Farrah et al. highlight the potential of “retinal fingerprints” as dynamic biomarkers, leveraging DL to link retinal vascular features with patient-specific cardiovascular profiles, which could revolutionize precision medicine by integrating imaging data into real-time risk stratification. As imaging hardware improves and ML models mature, retinal fingerprinting is poised to transform both high-security identification and personalized healthcare [57].

AI predicting systemic diseases: current tools and evidence

Cardiovascular Diseases​​​​​

CVDs remain the leading cause of death across the globe, resulting in 20.5 million deaths in 2021 [58], emphasizing the importance of early and accurate identification of risk. The traditional CVD risk scores (e.g., Framingham, QRISK3) incorporate established risk factors but often fail to capture the risk associated with subclinical disease [59]. AI tools that use retinal imaging can significantly improve CVD predictions and outperform traditional methods. Below, we summarize current AI tools supported by clinical evidence.

RetiAGE: RetiAGE is a DL algorithm that analyzes retinal images to estimate the retina's biological age, which has been shown to effectively stratify the risk of mortality and cardiovascular events [60]. In the UK Biobank study, participants in the highest RetiAGE quartile had a 142% increased risk of CVD mortality and a 39% increased risk of a CVD event over a 10-year period compared with those in the lowest RetiAGE quartile, even after adjusting for chronological age and other known biological aging measures. RetiAGE exhibited strong performance in predicting CVD mortality, with a c-index of 0.70, sensitivity of 0.76, and specificity of 0.55, and its inclusion improved the model’s discriminative ability beyond traditional measures [61].

DL Funduscopic Atherosclerosis Score (DL-FAS): DL-FAS is a score derived from retinal fundus images evaluated using a DL model developed by Chang et al. in 2020, which indicates the degree of atherosclerosis. DL-FAS was then tested in a cohort of over 32,000 adults, with a median follow-up of 7.6 years. The results showed that individuals with a high DL-FAS (>0.66) had a significantly higher risk of cardiovascular death than those with a low score (<0.33), with a hazard ratio (HR) of 8.33 (95% confidence interval (CI) = 3.16-24.7). This model achieved an area under the curve (AUC) of 0.713 [62].

Multi-channel variational autoencoder (MCVAE): Diaz-Pinto et al. developed an AI model based on an MCVAE that uses retinal images to predict cardiovascular risk [63]. The model was trained using paired datasets of retinal fundus photographs and corresponding cardiac MRI measures for left ventricular (LV) mass and volume, using only retinal images. LV mass and volume estimates are used to predict the probability of future myocardial infarction (MI) or heart attack. The authors found that the retina-based model achieved an AUC of 0.80 for predicting incident MI, with a sensitivity of 74% and a specificity of 71%, leveraging only retinal images and basic demographic information [64].

Google DeepMind’s retinal risk prediction: The study by Poplin et al. at Google DeepMind is a breakthrough in using DL methods to predict cardiovascular risk factors from a standard retinal fundus image. The model was trained on data from 284,335 patients and learned to predict a range of patient characteristics and risk factors that are not visible through classical eye screening. The DL model was able to estimate a person's age (±3.3 years), sex (AUC = 0.97), smoking status (AUC = 0.71), and blood pressure (±11 mmHg). The model also accurately predicted the probability of future major adverse cardiovascular events, such as MI or cerebrovascular events, with an AUC of 0.70 [65]. The algorithm generates predictions by analyzing the anatomical structure of the eye, particularly the optic disc and retinal vasculature; the attributes of these structures, including vessel diameter and tortuosity, provide significant insights into vascular health [66].

VUNO Med-Fundus AI™: The VUNO Med-Fundus AI tool was developed by Son et al. for automated screening of multiple retinal abnormalities in fundus photographs. The VUNO model was trained on over 309,786 readings from 103,262 images and can identify 12 key retinal findings, including hemorrhages, hard exudates, cotton-wool spots, drusen, macular holes, retinal nerve fiber layer defects, and optic disc changes (both glaucomatous and non-glaucomatous), among others. The results of internal validation were remarkable, with AUC for system performance ranging from 96.2% to 99.9% for different findings. The system maintained an AUC of approximately 94% to 98% for DR-associated lesions on external datasets and, for many other findings, achieved performance levels on par with those of expert ophthalmologists [67]. By analyzing a wide array of retinal signs, this tool can indirectly flag systemic issues; detecting hypertensive retinal changes could prompt a cardiovascular risk evaluation. A study showed that using the VUNO tool improves diagnostic accuracy, facilitates agreement among doctors, and reduces reading time, especially for less experienced readers and simpler retinal images [68]. The VUNO Med-Fundus AI tool received regulatory approval from MFDS as a Class III medical device [69].

Retinal Cardiovascular Disease Risk Score (Reti-CVD): The Reti-CVD is an AI tool that predicts the Reti-CVD score using retinal images and CT-measured coronary artery calcium (CAC). In a key experiment using data from the Cardiovascular and Metabolic Diseases Etiology Research Center-High Risk, Reti-CVD stratified participants into low-, moderate-, and high-risk groups, with increasing risk stratification strongly associated with a higher five-year incidence of CVD events. Based on these results, Reti-CVD received approval from the Korean regulatory body [70]. The Reti-CVD was also validated in the UK Biobank, demonstrating its usefulness in identifying patients with a 10-year CVD risk of 10% or more, particularly those with borderline QRISK3 scores. The results indicate that Reti-CVD has the potential to be a valuable, noninvasive means of improving cardiovascular risk stratification across various populations [71].

Diabetes and Related Complications

The application of autonomous AI systems in ophthalmology has revolutionized the timely diagnosis and treatment of DR, a major contributor to visual impairment among adults. Several AI-driven diagnostic systems have been established and deployed worldwide, with high accuracy and efficiency reported in DR screening. Below is a summary of current AI tools supported by clinical evidence.

LumineticsCore™ (formerly IDx-DR): Digital Diagnostics' LumineticsCore™, an independent DL system, is designed to detect conditions beyond mild DR and DME by analyzing retinal fundus images. In a key multicenter primary care study, it yielded 87.2% sensitivity and 90.7% specificity for referable DR [72]. It was the first AI system to meet pre-specified Food and Drug Administration (FDA) endpoints in a prospective study. It received FDA approval in 2018 as the first standalone AI diagnostic in any field [73]. The system also received the CE Mark (class IIa) in Europe.

EyeArt®: Developed by Eyenuk, EyeArt® is an autonomous DL system for detecting more-than-mild DR (mtmDR) and vision-threatening DR (vtDR). A prospective multicenter trial involving 942 patients demonstrated approximately 96% sensitivity and 88% specificity for mtmDR and 92% sensitivity and 94% specificity for vtDR. The system achieved 97% imageability without the need for dilation in 90% of eyes [44]. EyeArt received FDA 510(k) clearance in 2020, making it the first to detect both moderate and vtDR in a single test [74].

AEYE-DS: AEYE-DS, developed by AEYE Health, is an autonomous AI system optimized for referable DR detection using portable, handheld fundus cameras. Two Phase III studies demonstrated approximately 92-93% sensitivity and 89-94% specificity in detecting referable DR. The system exhibited extremely high imaging success, with over 99% of patients receiving a result, indicating robustness even with non-mydriatic portable cameras. AEYE-DS became the first autonomous DR AI approved by the FDA for handheld camera use. This product can be purchased with either a tabletop or portable fundus camera. It is the only product on the market that requires a single image for screening of each eye. This feature reduces patient screening time to less than one minute [75].

SELENA+: SERI and EyRIS have developed the SELENA+ application, which uses DL technology to identify DR cases and can also detect glaucoma and AMD. Implemented within the Singapore Integrated Diabetic Retinopathy Program (SiDRP), SELENA+ significantly improved screening coverage and capacity. A prospective analysis of 1,712 consecutive patients in SiDRP revealed a sensitivity of 94.7% and a specificity of 82.2%. Compared with human graders, SELENA+ showed higher sensitivity for identifying vtDR with fine-tuning, though overall sensitivity and specificity were higher among human graders [76]. In October 2019, SELENA+ received regulatory approval from the Singapore Health Science Authority, the European CE Mark in March 2020, and the Medical Device Authority of Malaysia in June 2020 [77].

DAPHNE: DAPHNE is a computer vision-based retinal image analysis program developed by the University of Surrey to improve DR screening and disease progression tracking. It was developed using CNNs and uses the UK National Screening Committee grading system and the International Clinical Diabetic Retinopathy Severity Scale to classify DR severity. Retinal structures and lesions, such as microaneurysms, hemorrhages, exudates, intraretinal microvascular abnormalities, and neovascularization, are detected by the system using U-Net and CNN-based detectors. When DAPHNE was evaluated across a variety of populations in China, Saudi Arabia, and Kenya, it demonstrated a sensitivity of 97.1% and a specificity of 95.6% for detecting DME and referable DR. DAPHNE provides lesion visualization with supporting evidence and allows the monitoring of the progression of DR over time, thereby facilitating clinical decision-making [78].

Neurological Conditions

Recent studies indicate that retinal imaging and ML together constitute a novel noninvasive technique for identifying individuals at risk of neurodegenerative disorders. One such study proposed a trilateral AI model that utilizes OCT imaging to determine whether an individual is cognitively normal, has mild cognitive impairment, or is diagnosed with AD [79]. The model combined three streams: the first used a CNN for RNFL maps, the second used a Swin Transformer for GC-IPL maps, and the third utilized a Naive Bayes classifier for OCT features. XGBoost was used for the final prediction. During external validation, the model demonstrated excellent diagnostic performance, with AUCs of 0.91 (95% CI, 0.79-0.98) for the Asian population and 0.84 (95% CI, 0.76-0.90) for the White population [79]. Radiant’s model also outperformed traditional OCT metrics in both populations, achieving 88% sensitivity and 82% specificity in the Asian population and 67% sensitivity and 71% specificity in the White population [79].

The DeepRETStroke study used a DL system to detect silent brain infarctions and predict future stroke events from retinal imaging. The primary model was developed on a robust dataset of 895,640 retinal images and subsequently validated on 213,762 retinal images from various international populations, including China, Singapore, Malaysia, the United States, the United Kingdom, and Denmark. The model’s AUCs for predicting future and recurrent stroke were 0.901 and 0.769, respectively [80]. When applied to these cohorts, the model demonstrated external validation by maintaining strong performance. Further, it outperformed traditional clinical risk factors in determining secondary prevention strategies [80].

In parallel, the RETFound model represents a novel foundation model trained on 1.6 million unlabeled retinal images (fundus photography and OCT) using a masked image reconstruction task [81]. Fine-tuned on minimal labeled data, including dementia-related annotations, it demonstrated superior performance over traditional models such as SL-ImageNet [81]. By identifying subtle retinal biomarkers of cognitive decline, RETFound provides a scalable method for dementia risk stratification.

Renal Disease

Researchers at the Singapore Eye Research Institute developed RetiKid, a DL model capable of predicting CKD from retinal fundus photographs alone, leveraging the concept that retinal microvasculature mirrors systemic microvascular pathology, including in the kidneys [82]. Using over 25,000 images from the Singapore Epidemiology of Eye Diseases study, a CNN was trained to identify individuals with CKD (defined as eGFR < 60 mL/min/1.73 m²). Three model configurations were tested: (i) an image-only model, (ii) a clinical-only model (age, sex, diabetes, and hypertension), and (iii) a model combining both modalities. During internal validation, the image-only model achieved an AUC of 0.911 [82]. Moreover, the AUCs of 0.733 and 0.835 in SP2 (Singapore) and the Beijing Eye Study (China), respectively, indicate that the model generalizes across two independent external validation cohorts [82].

**Figure 2:**
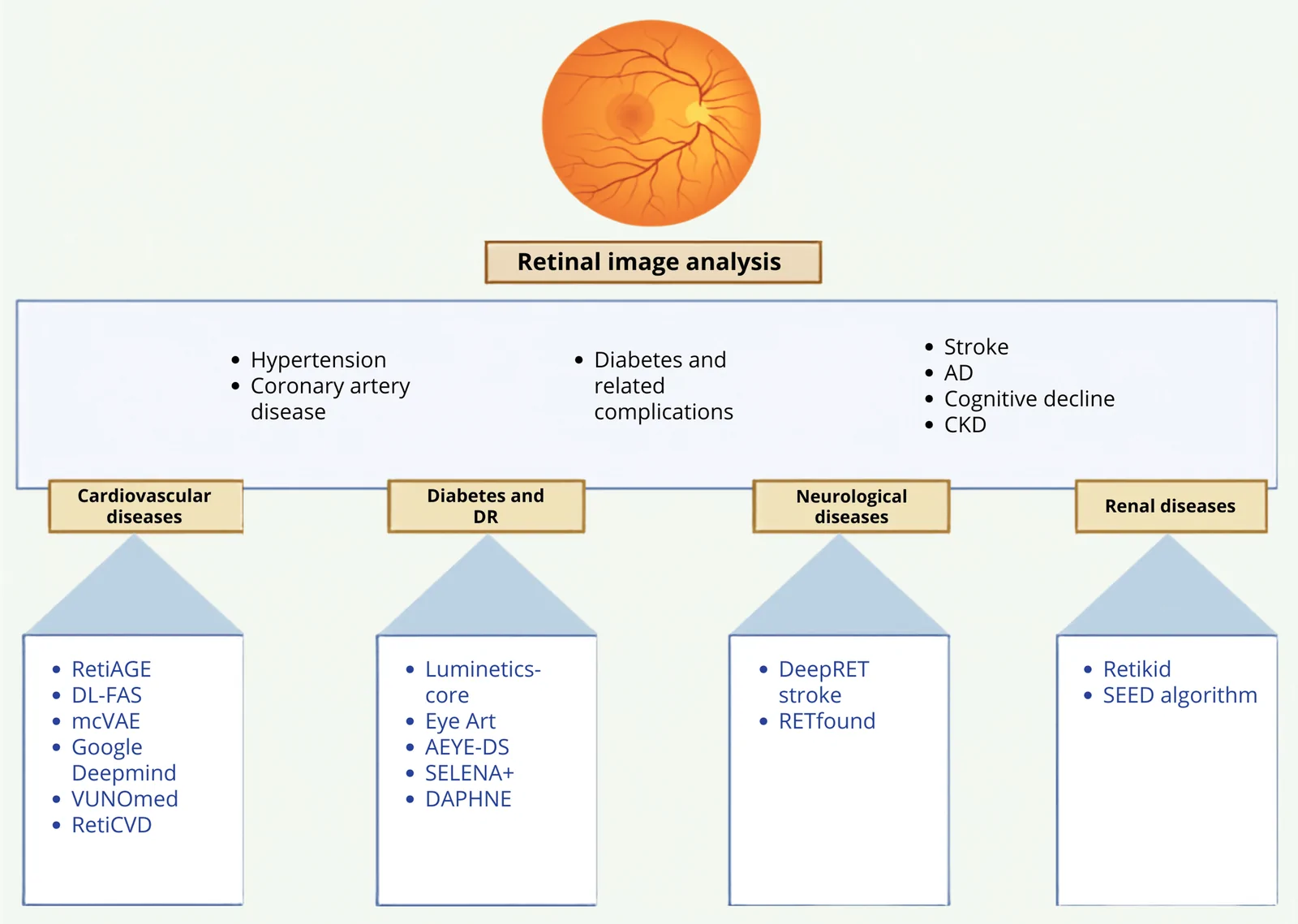
AI algorithms for systemic disease detection AI: artificial intelligence, DR: diabetic retinopathy, CKD: chronic kidney disease, AD: Alzheimer’s disease Image Credit: Kauser T (2025) using Science Suite Inc. (BioRender; Toronto, Ontario, Canada)

Although the hybrid model outperformed the individual models, the image-only model also performed strongly on its own. This indicates that retinal imaging could serve as a low-cost, noninvasive tool for large-scale CKD screening, especially in settings where blood testing is impractical. Notably, the model functioned without requiring the AI to explicitly recognize individual retinal features. Rather, it learned latent vascular patterns associated with renal impairment. Subsequent studies validated RetiKid in diabetic cohorts [83] and demonstrated its utility for risk stratification, outperforming traditional risk scores in predicting incident CKD (Figure 2) [84]. Table 1 summarizes AI-based retinal imaging algorithms for systemic disease screening, highlighting their clinical applications, key advantages, and current challenges.

**Table 1:** AI-based retinal imaging algorithms for systemic disease screening: clinical applications, advantages, and challenges AD: Alzheimer’s disease, AMD: age-related macular degeneration, AUC: area under the curve, BP: blood pressure, CAC: coronary artery calcium, CKD: chronic kidney disease, CVD: cardiovascular disease, DL-FAS: deep learning funduscopic atherosclerosis score, DME: diabetic macular edema, DR: diabetic retinopathy, FDA: Food and Drug Administration, MACE: major adverse cardiovascular events, MCI: mild cognitive impairment, MI: myocardial infarction, MRI: magnetic resonance imaging, mtmDR: more-than-mild diabetic retinopathy, OCT: optical coherence tomography

Disease condition	AI algorithm	Clinical application	Advantages	Challenges
CVD	RetiAGE	Estimates biological age from retinal images to stratify CVD risk [61]	Improves CVD risk prediction beyond traditional scores; high sensitivity	Lower specificity; dependent on image quality [61]
	DL-FAS	Predicts atherosclerosis and cardiovascular death risk from fundus images [62]	Strong risk stratification; validated over 32,000 adults [62]	Needs further validation in diverse populations
	mcVAE	Predicts MI risk using retinal images and estimated cardiac MRI parameters [63]	High AUC (0.80); noninvasive and efficient [64]	Limited to paired datasets; generalizability unknown
	Google DeepMind Model	Predicts CVD risk factors and MACE events from fundus images [65]	Can predict multiple risk factors (age, BP, smoking, etc.); large training dataset [66]	Model complexity; interpretation transparency
	VUNO Med-Fundus AI™	Screens for retinal signs indirectly linked to systemic diseases [67]	High AUC (96-99%); supports physician agreement; speeds up diagnosis [68]	Indirect prediction of CVD; tool used mainly for retinal findings [69]
	Reti-CVD	Predicts CVD risk using retinal image and CAC data [70]	Validated in UK Biobank and CMERC; noninvasive; regulatory approved [71]	Requires CAC data; external validation still emerging [71]
Diabetes and DR	LumineticsCore™ (IDx-DR)	Detects referable DR and DME in primary care settings [72]	FDA-approved; autonomous; 87.2% sensitivity, 90.7% specificity [73]	Needs high-quality images; no lesion grading [73]
	EyeArt	Detects mtmDR and vtDR without dilation [44]	FDA-cleared; >95% sensitivity; works without dilation in 90% of eyes [74]	Requires fundus camera; needs follow-up systems
	AEYE-DS	Detects referable DR with handheld cameras [75]	FDA-cleared; very high imaging success; quick (under 1 min)	May vary with operator skill; relies on image clarity [75]
	SELENA+	Detects DR, glaucoma, and AMD [76]	Regulatory-approved; improved screening capacity in Singapore [77]	Slightly lower specificity than human graders
	DAPHNE	Classifies DR severity and monitors disease over time [78]	97.1% sensitivity, 95.6% specificity; supports disease progression tracking [78]	Complexity of grading systems; population diversity in validation needed
Neurological disease	Trilateral AI model (VGG-11, Swin Transformer, Naïve Bayes, XGBoost)	Classifies cognitive state (normal, MCI, AD) from OCT scans [79]	High AUC (0.91 Asian; 0.84 white); multimodal input increases accuracy	Variability in OCT devices; White cohort had lower sensitivity [79]
	DeepRETStroke	Detects silent brain infarctions and predicts stroke risk [80]	Very high AUC (0.901); validated on large multinational datasets	Requires vast and diverse datasets for training [80]
	RETFound	Foundation model for dementia risk via retinal biomarkers [81]	Scalable; pretrained on 1.6M images; needs minimal labels	Still under development; may need local fine-tuning [81]
Renal disease	RetiKid	Predicts CKD from retinal fundus images [82]	High AUC (0.911); validated externally; low-cost and noninvasive [83]	Limited specificity to CKD subtypes; blood test confirmation still standard

Integrative AI models and multimodal data fusion

Multimodal data fusion combines results from various retinal imaging techniques and integrates laboratory findings, genetic data, and clinical measures. This approach has improved retinopathy classification and enhanced the accuracy of systemic disease prediction [85]. Disease identification may be improved by combining exposure history, metadata, electronic health records, and results from various imaging modalities [14].

A study conducted in France from 2020 to 2022 across 14 hospitals, involving 875 eyes from 444 patients, found that a multimodal analysis combining results from ultra-widefield CFPs (UWF-CFP) and OCTA improved DR classification. The multimodal analysis achieved an AUC score of 0.8566 for mild non-proliferative DR. This score was higher than the scores of 0.7983 for UWF-CFP and 0.8316 for OCTA when used individually [86]. OCT and retinal fundus photography have been used together to detect glaucoma [87]. Age-related macular degeneration is diagnosed using OCT, OCTA, and retinal fundus photography [88].

HyMNet is an AI-driven technique that integrates retinal imaging data with electronic health record information, including factors such as age and gender, to predict the risk of CVD and hypertension. A study conducted in Saudi Arabia, involving 1,243 fundus photographs, reported an F1 score of 0.771 with HyMNet [89].

A case-control study was conducted at Samsung Medical Center, where data from fundus photographs were combined with health record information, including risk factors such as age, systolic blood pressure, diabetes, and hypertension, to predict the occurrence of heart diseases. The AUC was 0.781. This study showed that combining multiple modalities with AI assistance improves the prediction and prognosis of CVDs [90]. Radiological reports and retinal imaging contain information that can be used to evaluate the risk of systemic diseases. One approach involves using multimodal bidirectional long short-term memory networks to incorporate information from radiology notes [91].

RetiPhenoAge represents a potential advance in the early ascertainment of potentially life-threatening illnesses. RetiPhenoAge incorporates a proprietary AI module to synthesize retinal imaging along with molecular components such as telomeres and handgrip strength. This technology has the potential to correlate retinal changes with anomalies in the kidneys, liver, heart, and immune system. RetiPhenoAge has been reported to significantly assist with early detection of cancer and cardiovascular events [92].

Clinical applications and ethical considerations

The introduction of AI technologies in ophthalmology is expected to enable more precise diagnoses and improved clinical workflow. Additionally, there are concerns about privacy and data security [93]. Certain AI applications focus on integrating and analyzing diverse patient datasets. An example is the integration of cross-sectional data, including the patient's birth date, genetic data, images, and their therapeutic history. This analysis contributes to generating highly specific, individualized predictions to enhance the quality of clinical decisions [94-96].

AI-assisted technology has the potential to accelerate pathways to direct access to care by systematically identifying and addressing the backlog of referral cases. Strategic deployment of this technology will enable stakeholders in healthcare services to prioritize referrals in areas of greatest need and improve the cost-efficiency of healthcare services [97].

**Figure 3:**
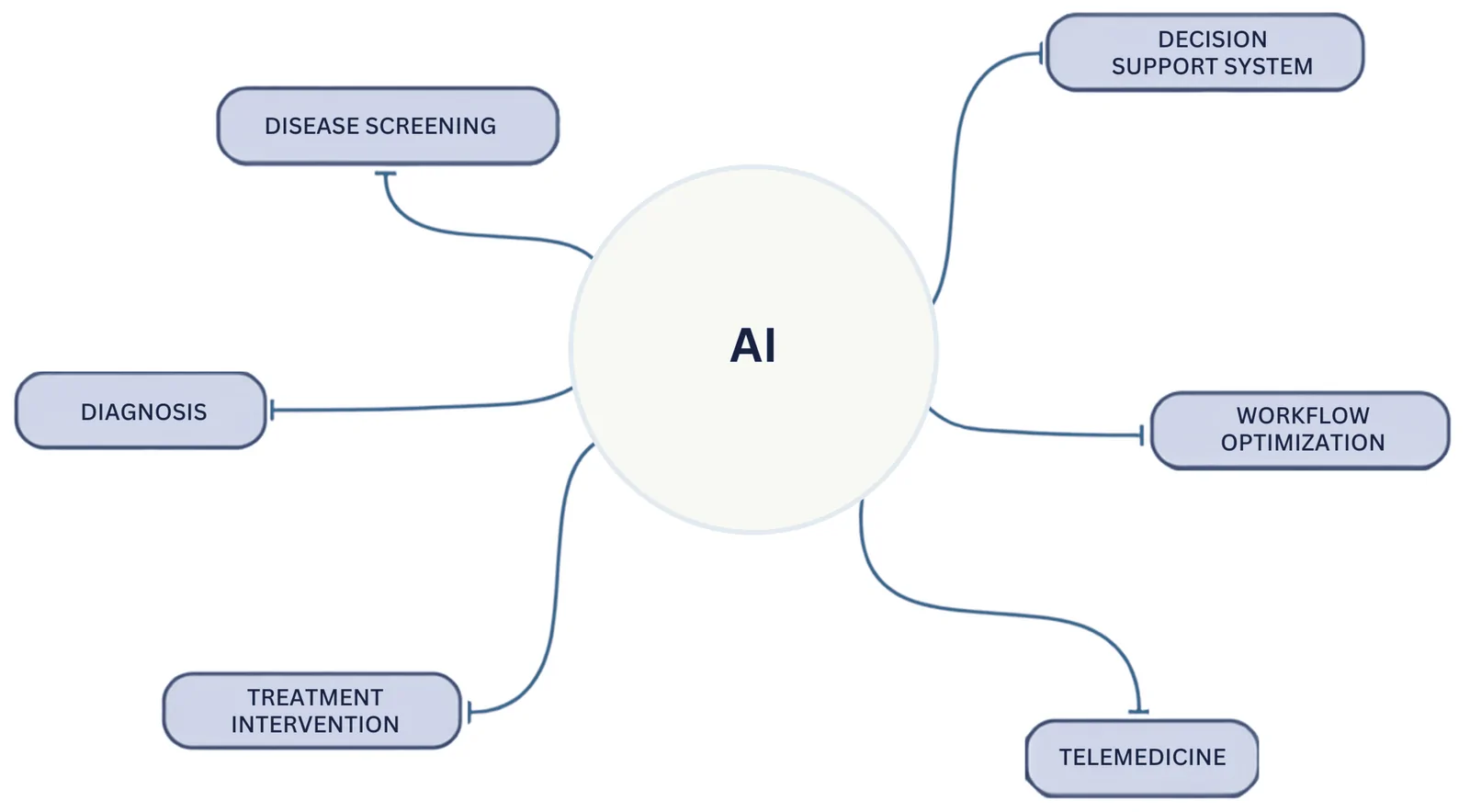
AI services in precision medicine AI: artificial intelligence Image Credit: Kauser T (2025) using Science Suite Inc. (BioRender; Toronto, Ontario, Canada)

Telemedicine enables patients to obtain medical care remotely using a variety of technologies, including asynchronous messaging, scheduled video calls, and other applications. Essentially, telemedicine enables communication with clinicians, including but not limited to primary care providers and specialists, from any location that best serves the patient [98]. In 2021, 25% of visits to the ophthalmologist were conducted over telemedicine, increasing patient access to care. Among patients seen in person, most required specialized procedures due to effective triage by optometrists. The use of videoconferencing enabled more patients to be added to the surgical waiting list, thereby reducing waiting time and the associated cost and anxiety (Figure 3) [99].

Programs in Australia and Nepal, such as the Lions Outback Vision program and community-based models, respectively, have demonstrated the ability of DL algorithms to accurately diagnose eye diseases and to provide eye care in remote and underserved areas [98]. Mobile AI technologies, exemplified by EyeArt and Google Health's DR model, use smartphone-based fundus cameras to enable rapid, precise DR screening. These technologies are currently deployed in community clinics and outreach programs, improving the capacity for early case detection in resource-limited settings [96,100].

AI technologies have the potential to be deployed most effectively in areas beyond the reach of conventional eye care. Portable devices incorporating AI technology and cloud-based screening systems have been deployed in rural and Indigenous communities to facilitate the early detection and management of retinal diseases without the need for a specialist [97,98]. These advances contribute to fair access to healthcare by addressing geographic and/or structural barriers. The absence of open-source tele-ophthalmology solutions creates a substantial barrier to the development and deployment of large-scale eye care technology solutions in low-income countries. There are many barriers to adoption, ranging from monetary issues and skepticism toward new technology to social barriers, structural deficiencies, and concerns about reliability and usability [100].

Black box: A challenge, popularly termed the "black box phenomenon," is the limited interpretability of an AI system's internal processes. This occurs when data is input into the model and an output is produced without a clear descriptive method for the process. Transparency is a critical challenge for many AI applications. With the black box phenomenon, the reasoning behind the outputs is invariably concealed [101]. Clinician trust and patient confidence are both adversely affected by the absence of interpretability. Explainable AI (XAI) techniques, such as saliency maps and class activation visualizations, attempt to mitigate the gap but remain largely absent from clinical workflow integration [102].

Generalizability: Generalizability is a major challenge in evaluating the progression of AI applications in ophthalmology and their implementation for the public. As AI models using large datasets are developed to improve accuracy, it is essential to consider diverse races and ethnicities to ensure comprehensive population coverage. This was retrospectively reviewed by Cheung et al., who trained a DL model to detect AD. Their model provides a unique and generalizable framework that could be used in community settings and across diverse populations [103]. However, large model datasets are required to improve generalizability and to enhance the accuracy of DL models [104].

Bias: Another aspect to consider is the potential bias in AI model outcomes. Bias can arise from the limited demographic and ethnic composition of the training dataset. The goal is for the AI model to consistently perform the intended function without, or at least with limited, hidden biases. This approach is of significant importance to clinicians utilizing AI systems [105]. Solutions include low-shot learning, generative DL to synthesize balanced datasets, and subgroup-specific performance auditing during model development [102].

FDA-approved AI architectures: With the advent and ongoing advancement of AI systems in ophthalmology, there is great emphasis on integrating these systems into clinical practice following FDA approval. One of the earliest integrated systems is the 2018 autonomous DR and DME detection software (IDx-DR) [72]. Another AI system subsequently received FDA authorization for clinical screening in 2020 [106].

Public and clinician trust: Despite such advancements, AI adoption continues to face challenges due to public concerns about data privacy. Clinicians, on the other hand, seek to build trust when using AI modules to formulate diagnoses. The goal is to create personalized management pathways based on predictive analyses [107]. AI-based detection and integration may therefore play an increasingly important role in clinical care [108].

Building Trust Through Explainable AI

XAI plays a vital role in building trust among clinicians and patients. Visual tools that highlight areas of interest on retinal images help clinicians validate outputs and understand model behavior [102]. Transparent models enable shared decision-making and patient reassurance, particularly in tele-ophthalmology, where face-to-face interaction is limited. In regulatory and clinical validation contexts, XAI contributes to auditability, model debugging, and safety assurance. The integration of XAI into telehealth platforms is expected to become a requirement for widespread adoption and clinical trust [97].

Research gaps and future directions

Looking ahead, the accelerated development of AI for retinal diagnostics necessitates a strategic transition towards global inclusivity, standardized data, and real-world validation, which will be essential to improving healthcare standards. Moreover, there is a risk that the underrepresentation of minorities and global populations in training datasets for AI systems will perpetuate systemic bias in both diagnosis and treatment. AI models, especially those developed in high-income nations and trained on homogeneous datasets, overlook the diverse genetic, environmental, and socioeconomic determinants prevalent in underrepresented regions, such as Latin America and Sub-Saharan Africa [109,110]. For AI models to perform equally across demographics, it is of utmost importance that they be trained and validated on datasets derived from diverse retinal phenotypes [111]. Additionally, the lack of standardized datasets and labeling strategies across institutions hinders reproducibility and limits the broader applicability of AI tools [112]. The development of international open-source platforms that standardize annotation practices will be crucial to ensuring that AI diagnostic systems are not only accurate but also scalable, validated, and clinically trustworthy across different geographical regions [113].

**Figure 4:**
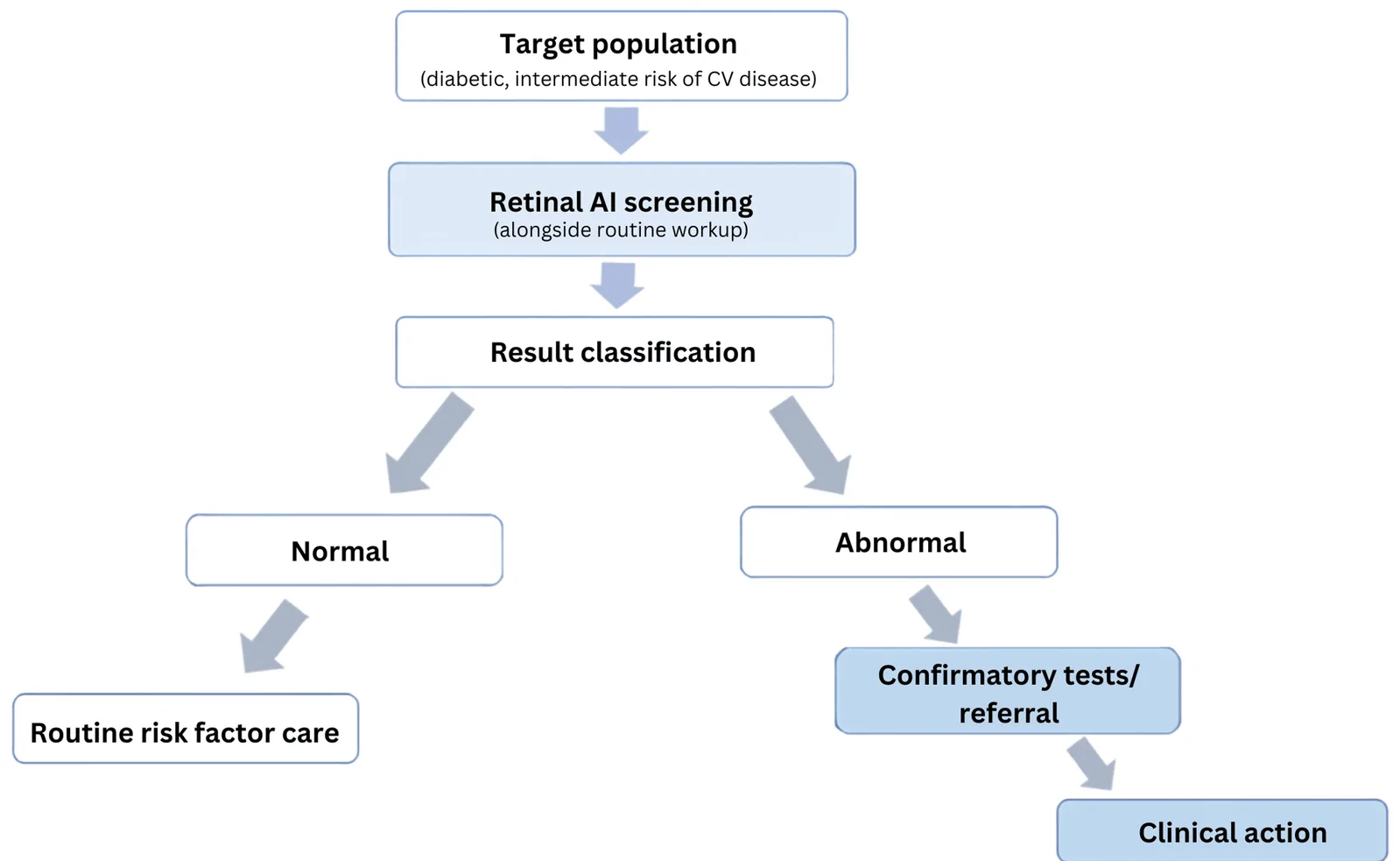
Future directions in AI-based retinal imaging AI: artificial intelligence, CV: cardiovascular

Furthermore, integrating AI-based retinal biomarkers with wearables and point-of-care technologies has the potential to drive significant breakthroughs in global health. The combination of portable retinal imaging and AI has the potential to enable accessible, early, and noninvasive screening for systemic pathologies, including stroke, CKD, and neurodegenerative conditions, in resource-limited environments where access to specialized healthcare is severely restricted [104,110,114]. Moreover, recent advances in wearable biosensors and compact point-of-care devices for CKD monitoring highlight the growing potential to link retinal AI diagnostics with continuous, accurate, real-time health monitoring outside of traditional clinical environments [115]. Despite these innovations, their success largely depends on addressing current research deficits, such as the lack of longitudinal validation studies and an overreliance on training AI models using cross-sectional analyses that fail to capture the dynamic nature of disease progression (Figure 4) [14,116]. Future research should focus on combining AI retinal biomarkers with continuous, real-time monitoring technologies and large, diverse longitudinal studies to develop clinically accurate, validated diagnostic systems that facilitate early intervention and personalized care. The combination of AI technology, mobile diagnostics, and diverse imaging has the potential to shape the future landscape of global precision medicine [117].

## Conclusions

Retinal imaging combined with AI offers a noninvasive means of visualizing retinal blood vessels and nerves, enabling detection of early, subclinical signs of systemic disease. Rather than replacing established assessments, retinal AI may offer incremental value by identifying microvascular and neurodegenerative changes that precede or accompany abnormalities detected by conventional measures, potentially enabling earlier risk stratification in individuals who otherwise appear to be at low or intermediate risk. For example, in patients with borderline or intermediate cardiovascular risk scores, an abnormal retinal AI finding could prompt closer monitoring, earlier initiation of risk-factor control, or referral for further systemic workup, rather than serving as an isolated imaging-based diagnosis.

However, current evidence is largely limited to associations between retinal biomarkers and disease risk, with few studies demonstrating that AI-guided retinal intervention improves clinical outcomes beyond conventional risk assessment. Defining clear screening populations, actionable result thresholds, and downstream referral pathways will be necessary before retinal AI can be meaningfully integrated into clinical decision-making.

The integration of technological advances into clinical practice may improve population health. AI-based retinal imaging is a promising tool for the early detection of a wide range of vascular, metabolic, and even neurological conditions. The continued evolution of this imaging technique may yield novel advances in preventive medicine.
